# The role of sex-differential biology in risk for autism spectrum disorder

**DOI:** 10.1186/s13293-016-0112-8

**Published:** 2016-11-16

**Authors:** Donna M. Werling

**Affiliations:** Department of Psychiatry, University of California, San Francisco, San Francisco, CA 94143 USA

**Keywords:** Autism spectrum disorder, Autism, Sex differences, Prevalence, Female protective effect, Testosterone

## Abstract

Autism spectrum disorder (ASD) is a developmental condition that affects approximately four times as many males as females, a strong sex bias that has not yet been fully explained. Understanding the causes of this biased prevalence may highlight novel avenues for treatment development that could benefit patients with diverse genetic backgrounds, and the expertise of sex differences researchers will be invaluable in this endeavor. In this review, I aim to assess current evidence pertaining to the sex difference in ASD prevalence and to identify outstanding questions and remaining gaps in our understanding of how males come to be more frequently affected and/or diagnosed with ASD. Though males consistently outnumber females in ASD prevalence studies, prevalence estimates generated using different approaches report male/female ratios of variable magnitude that suggest that ascertainment or diagnostic biases may contribute to the male skew in ASD. Here, I present the different methods applied and implications of their findings. Additionally, even as prevalence estimations challenge the degree of male bias in ASD, support is growing for the long-proposed female protective effect model of ASD risk, and I review the relevant results from recurrence rate, quantitative trait, and genetic analyses. Lastly, I describe work investigating several sex-differential biological factors and pathways that may be responsible for females’ protection and/or males’ increased risk predicted by the female protective effect model, including sex steroid hormone exposure and regulation and sex-differential activity of certain neural cell types. However, much future work from both the ASD and sex differences research communities will be required to flesh out our understanding of how these factors act to influence the developing brain and modulate ASD risk.

## Background

Autism is a lifelong neuropsychiatric condition first apparent during early development that is characterized by social and communication deficits and by repetitive behaviors and restricted interests. The severity and variety of symptomatic behaviors, impairments, and abilities that autistic individuals show is vast, leading to the formal conceptualization of autism as a spectrum (autism spectrum disorder, ASD) [1]. ASDs also differ by sex, with a striking and consistent male bias in prevalence [2, 3].

In recent years, interest in investigating sex differences in the autistic phenotype and exploring a potential need for sex-differential diagnostic criteria has grown more widespread [4, 5]. At the same time, research findings and public discourse have challenged the magnitude of the male bias in prevalence [6–10], and genetics studies have demonstrated patterns of risk variation that are consistent with a protective effect against the ASD phenotype in females [11–21]. Work to identify the sex-differential factor(s) responsible for this protection has returned several potential leads, but the key factor(s) involved remains unknown, and the molecular, cellular, and/or neurodevelopmental pathway(s) by which these factors impact risk are not currently understood. Given the strong impact of sex on ASD prevalence and/or presentation, understanding the points of interaction between sex-differential factors and ASD etiological pathways is likely to reveal critical aspects of ASD biology that may provide effective therapeutic targets. More and continued attention to these questions, particularly with input from the sex differences research community, is warranted to begin to make concrete sense of the ways that sex-differential neurodevelopment and brain function modulate neuropsychiatric risk. Here, I aim to summarize the current state of research findings on sex differences in ASD prevalence, phenotype, and risk mechanisms, as well as to highlight gaps in our current understanding that are likely to benefit from input from the sex differences research community.

### Autism prevalence is male-biased

The most striking sex difference in ASD is its prevalence, as approximately four times as many males have a diagnosis of ASD as females [2]. This 4:1 male:female ratio is a commonly cited statistic that represents a consensus across epidemiological studies conducted in different countries, at different times, and using different iterations of diagnostic criteria; on an individual study level, the degree of male skew can vary widely. Though recent in-depth prevalence studies have tended to report smaller male biases than the 4:1 estimate [6, 8, 10], ASD-diagnosed males consistently predominate across these and earlier epidemiological surveys [2, 3], making sex-biased prevalence one of the most temporally and geographically stable features of ASDs.

At face value, this pattern of disparate prevalence suggests the action of sex-differential risk factors for ASD that act to either increase males’ risk and/or protect females. Just a few decades ago, as our conceptualization of ASD shifted from the domain of psychoanalysis to neuropsychiatry and genetics, an assumption that sex-differential risk factors were also biological in nature followed suit [22, 23]. More broadly, this paradigm shift and the dismissal of parenting style as the cause of ASD (so-called “refrigerator mothers” [24, 25]) revealed gaps in our knowledge of autism that researchers have aimed to fill. For much of the field, top priority questions included characterizing the behaviorally defined autistic phenotype in neuroscientific terms, particularly from the cognitive neuroscience [26, 27] and structural/functional neuroanatomical perspectives [28–31], with the intention to leverage these descriptions to discover ASD’s underlying causes. During this time, a handful of research groups published studies that compared males and females with ASD on the presentation and severity of their autism symptoms [32–35] or on neuroanatomical features [36, 37]. However for a majority of analyses, despite the male skew in ASD’s prevalence, sex was most frequently considered a variable to control for, not an aspect of risk to investigate in its own right. Often, to reduce experimental variability, characterization studies of autistic behavior, cognition, neuroimaging, and neuroanatomy only included male participants with ASD.

Still, despite more widespread focus on characterizing the autistic phenotype and its cause(s), the hypothesis that some aspect of male and/or female biology modulates ASD risk remained. Several research groups proposed the involvement of general sexually dimorphic factors such as X-linkage [38, 39], imprinting [40, 41], and sex steroid hormone levels [42]. However, another, non-mutually exclusive possibility is that females *are* affected by ASD at higher rates than previously thought, but that they are not being diagnosed. If this scenario were true, it would require a careful reexamination of the ASD phenotype, our understanding of which is based on the study of majority male cohorts, as well as our assumptions about sex-differential risk and protection for ASD.

Since males have predominated in studies of the features and phenotype of ASD, it can be argued that diagnostic criteria and instruments for ASD preferentially describe what ASD looks like in a male. The manifestation of ASD in females, then, may not appear to meet diagnostic criteria, which would lead to a smaller number of females being diagnosed and an apparent male bias in prevalence. To better understand the female’s autistic phenotype, studying diagnosed females is informative, but results must be interpreted in light of the caveat that these are the females who are identifiable under potentially male-biased diagnostic criteria. Additional work is required to unpack the possibility and consequences of missed diagnoses in females.

Ideally, such work should explore both (1) phenotypic traits in non-diagnosed females and (2) long-term outcomes. In particular, evaluation of females without ASD diagnoses who meet at least a subset of current defining criteria and/or who have other neuropsychiatric or neurodevelopmental diagnoses may be informative for identifying potential gaps in diagnostic criteria where females are likely to fall short. Following such individuals longitudinally will also be required to determine if quality of life (e.g., lack of social engagement) and/or achievement outcomes (e.g., employment status relative to cognitive ability) are negatively impacted in these females. Findings of poor outcomes would indicate that these individuals stand to benefit from diagnosis and the services and therapies available to autistic patients. This would also motivate revision of the diagnostic criteria for ASD in order to better identify these females.

Detailed analyses of ASD prevalence have certainly hinted at the possibility of skewed diagnoses in males compared with females. For example, studies comparing autistic individuals across a range of intellectual ability have shown that the male bias is as high as 9:1 among cases with intelligence quotient (IQ) in the normal-to-high range (frequently termed “high functioning”) but as low as 1.6:1 among cases with intellectual disability [43–45]. Though intellectual ability or disability is not part of the diagnostic criteria for ASD, if intellectual ability is thought to reflect overall phenotypic severity, this pattern of fewer females among autistic patients with normal-to-high IQ could suggest that females are largely protected against all but the most penetrant risk factors.

Alternatively, it might be the case that females must present with comorbid intellectual disability or a clear syndrome in order to be evaluated for, or receive a diagnosis of, ASD. Given that our current understanding of ASD is based on a body of research from predominantly male cases, it has been suggested that either the diagnostic criteria for ASD, or clinicians’, educators’, and parents’ understanding of these criteria, do not accurately reflect how females present with ASD [4]. In this case, females’ ASD symptoms may tend to go unnoticed, particularly for high-functioning individuals with strong verbal skills, unless other troubling behaviors or difficulties prompt an in-depth evaluation [46]. A study of children with high levels of autistic traits who either met, or fell just short of, the diagnostic criteria for ASD found patterns consistent with this hypothesis: diagnosed girls were more likely than diagnosed boys to score significantly below average on a test of verbal and nonverbal cognitive ability and significantly above average on a measure of behavioral difficulties [47]. Similarly, a study of the distribution of quantitative ASD traits in families enrolled in a voluntary national registry found that a significantly smaller proportion of females than males with Social Responsiveness Scale (SRS) scores in the top 1% received ASD diagnoses from community professionals [48]. To determine the factors driving females’ versus males’ diagnoses, though, additional data will be required regarding the circumstances of each child’s diagnosis, including parents’ early concerns or motivation for seeking evaluation. Additionally, as the authors of the study of children above and below the diagnostic threshold caution, these findings could plausibly result from either gender-biased diagnoses or from protective mechanisms in females.

**Fig. 1:**
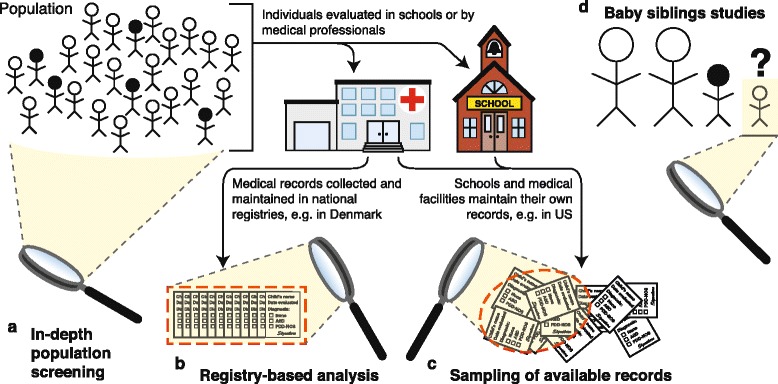
Approaches used to estimate ASD prevalence and male:female ratio. Different approaches may identify different overall, and sex-specific, prevalence rates. **a** Screening populations in full, irrespective of existing diagnostic status. **b** Analysis of records in existing, standardized registries. **c** Collating and/or sampling and interpreting available records from scholastic or medical records. **d** Baby siblings studies, where researchers prospectively monitor the younger siblings of autistic children for recurrence of ASD and other traits of interest. *Filled figures* represent individuals with ASD

Some degree of diagnostic bias is also evident in studies of ASD prevalence, as different methodological approaches uncover different male:female ratios among affected individuals. One common approach for estimating prevalence of a condition is to query existing records of diagnoses or symptoms. These records come from professionals in the community and incorporate these professionals’ interpretations and applications of the ASD diagnostic criteria (Fig. 1c). Prevalence studies using this record-based approach tend to report male:female ratios in the range of approximately 3:1 to 5:1 [2, 3, 49–54], the magnitude that is frequently cited. A second, more intensive approach is to screen a large sample of the general population for ASD traits, without *a priori* assumptions about which individuals are most likely to be affected (Fig. 1a). For example, instead of evaluating only children in special education classrooms for ASD, two recent large-scale studies in South Korea and Finland screened all school-age children in their selected samples [6, 8]. This unguided screening approach identified far more girls meeting criteria for ASD than record-mining studies typically do, with a 2.5:1 male:female prevalence in South Korea [6] and between 1.7:1 and 2.3:1 male:female ratio for different subsets of the autism spectrum in Finland [8]. In places where ASD screening is widespread, integrated into standard care, and diagnoses are recorded in government or private registries (Fig. 1b), similarly low male biases in prevalence have been reported, including 2.8:1 in Toyota, Japan [9], and 2.3:1 in the Stockholm Youth Cohort in Sweden [10].

On a smaller scale, another approach that allows researchers to thoroughly screen a sample for ASD without relying on records of community diagnoses involves prospective observation of the younger siblings of autistic individuals, who are at substantially elevated risk for ASD than the general population; this is often called a “baby sibs” study (Fig. 1d). One such study of Canadian children reported relative odds of ASD in male versus female siblings of autistic probands of only 1.65 [7]. An earlier study of children from 12 sites in the USA and Canada [55] and the largest study of these high-risk siblings to date [56] reported somewhat stronger male skews, with a relative risk of 2.8 and odds ratio of 3.18 (male versus female), respectively. However, the male biases in these studies are still on the lower end of reported sex ratios, together indicating that the high surveillance of these siblings, from investigators and parents, tends to identify a greater number of affected females.

Considered with full population and systematic screens, and in contrast to studies of diagnostic records, these patterns suggest that some number of affected females are not being diagnosed under the current system. One possible explanation for this is that, as described above, the current diagnostic criteria for ASD do not accurately describe the female presentation of ASD. Not only is this mismatch between criteria and presentation in females likely to impact estimates of ASD prevalence, but it likely has affected the ascertainment of samples and cohorts for studies as well. Therefore, this potential ascertainment bias toward males and strongly impacted females must be considered when interpreting reports of sex differences in ASD. It is still encouraging though that broad-based searches, in contrast to work on clinical records, identify a larger fraction of affected females using standard screening tools. This could suggest that it is not necessarily the diagnostic criteria themselves that are grossly male-specific. Instead, it could be that physicians’, teachers’, and parents’ interpretation of ASD symptoms in females may drive or exacerbate the male skew in prevalence.

If the results from unbiased population screens are any indication, we may find that increased awareness of the possibility of ASD in girls will subsequently facilitate an increase in the identification of affected individuals. For example, the Autism and Developmental Disabilities Monitoring Network (ADDM) of the Centers for Disease Control (CDC) in the USA periodically reports on ASD prevalence across multiple sites nationwide, and these reports show an increase in ASD prevalence over time [49, 57, 58]. A breakdown of ASD rates across time shows that a large contribution to this increase comes from diagnoses in school-aged males with milder ASD symptoms both in the USA [51] and in a Swedish cohort [10]. Study of incidence rates between 1995 and 2010 from the Danish Psychiatric Central Research Registry uncovered a comparable pattern of increasing incidence in older individuals (school age and above) and in milder subtypes of the autism spectrum including Asperger’s syndrome and Pervasive Developmental Disorder-Not Otherwise Specified (PDD-NOS) [59]. Increases in these specific subpopulations may reflect increased awareness of more subtle presentations of ASD, without comorbid intellectual disability.

Similarly, as awareness and understanding of ASD in females grow, we may begin to see increased rates of ASD in females reported by these prevalence-monitoring surveys. In fact, results from the study of incidence rates in Denmark demonstrate this very pattern, with increasing diagnoses in females leading to a reduction in the male/female ratio from 5.1 in 1995 to 3.1 in 2010; this reduced male bias was most striking for diagnoses of Asperger’s syndrome (8.4 to 3.0) and PDD-NOS (5.7 to 2.8) [59]. Additionally, results from the National Health Interview Study (NHIS) in the USA collected in 2014 show a 3:1 male/female ratio, down from 4.5:1 from the same survey in 2011–2013 [52]. Of note, the NHIS changed the order and format of its questions about ASD and developmental delay (DD) between the 2011–2013 and 2014 surveys such that the item on ASD was moved from a 10-condition checklist to a standalone question. In this new format, ASD status was queried before DD status. This formatting change may have contributed to the observed increase in ASD prevalence overall, from 1.25% in 2011–2013 to 2.24% in 2014, and a reciprocal decline in DD prevalence from 4.84% in 2011–2013 to 3.57% in 2014. Interestingly, in contrast to this overall decline in DD prevalence, the proportion of females with DD increased (34.6% in 2011–2013 to 36.7% in 2014), suggesting that males were mainly responsible for diagnostic substitutions between ASD and DD in the two survey periods. Therefore, the increase in the proportion of female children with ASD (18.3% in 2011–2013 to 25% in 2014) may also be at least partially attributable to the format change, but it is also possible that the observed increase in prevalence reflects increasing recognition of ASD in females. This recognition can only be accelerated by ongoing work to characterize females’ presentation and experience of ASD.

The recognition of shortcomings in our understanding of ASD in girls has sparked recent interest in studying autistic females, to better characterize the presentation of their symptoms, their cognitive and neuroanatomical phenotype, and how they differ from boys with ASD [4]. Thus far, studies of very young children have failed to identify sex differences in ASD symptoms among affected individuals [60, 61]. Outside of ASD-specific traits, one such study did also observe higher scores in affected females on the Daily Living Skills Subscale of the Vineland Adaptive Behavior Scales (VABS) [61]. Another study of male and female adults with ASD found no sex differences in retrospective reports of childhood autism traits, in keeping with the pattern above, but sex differences in social communication were apparent in adulthood [62]. Specifically, despite reports of equivalent ASD traits during childhood, adult females with ASD showed significantly fewer social communication difficulties than adult males during clinical evaluation (Autism Diagnostic Observation Schedule, ADOS).

Autistic females in this sample also self-reported higher scores on a measure of ASD symptoms compared with males, suggesting disconnect between their observed and experienced social behavior. This sort of behavior is consistent with the concept of “camouflaging” one’s ASD symptoms by making conscious, concerted effort to learn and emulate social norms. The application of this rote knowledge of social behavior may effectively hide an innate lack of skill in certain interactions, but the frequently monumental effort required to do this often goes unnoticed. In fact, though autistic females may have similar trouble with social communication as autistic boys do early in life, females may have greater social motivation and desire to be liked and engaged with her peers [62, 63]; this motivation may be what drives high functioning females in particular to camouflage their difficulties. It is unclear to what extent this desire and ability to compensate for social challenges by rote learning and performance of normative behaviors may be truly compensatory or protective against diagnosis in females, versus an exhausting and distressing burden for affected females to bear. It will be important to identify these girls who may escape diagnosis by engaging in camouflaging behavior and to determine their outcomes over time and the support they may need.

In addition to camouflaging behavior in high functioning females, studies of older children and adults with ASD are finding that autistic females tend to show reduced levels of restricted interests and repetitive behaviors compared with males. A large study of individuals from 970 families enrolled in the Autism Genome Project (AGP) observed lower repetitive behavior scores from the Autism Diagnostic Interview-Revised (ADI-R) in females [64]. A breakdown of the items contributing to the repetitive behavior scores further showed that this sex difference was driven by a reduction in females’ restricted interests but not of repetitive sensorimotor behaviors. Phenotypic characterization of 2418 cases in the Simons Simplex Collection (SSC) also revealed a similar pattern, with females showing reduced restricted interests [65]. Importantly, as female cases in the SSC are more likely to have cognitive impairment than male cases, IQ did not mediate this sex difference. Such reduction in restricted interests is also apparent in recent work, including a study of Australian children [63] and a study of autistic case data from the National Database for Autism Research (NDAR) and the Autism Brain Imaging Data Exchange Consortium (ABIDE) [66].

An alternative possibility is that females have just as many, or just as intense, restricted interests as males, but that these interests occur in different domains. In other words, autistic children’s interests may differ in much the same ways that the interests of male and female neurotypical children differ from one another, on average: while males with ASD might fixate on transit schedules or maps, females might fixate on horses or popular performers [67]. If diagnostic criteria more accurately represent the male phenotype, this may allow females’ restricted interests, as well as other phenotypic traits, to fly under the diagnostic radar [68, 69].

All together, a tendency toward increased social motivation, ability to consciously mask social impairment, and reduced or potentially non-prototypical restricted interests may cause affected females to not be evaluated or diagnosed [4, 70]. As characterization work continues and awareness of the possibility of ASD in females grows, we may find that diagnostic criteria and instruments need to be adjusted to better capture those girls who are struggling and would benefit from behavioral interventions and support. For example, the incorporation of female-typical exemplars into ASD diagnostic criteria and screening instruments (as has already been implemented in the Autism Spectrum Screening Questionnaire-Revised, ASSQ-REV [71]) might facilitate clinicians’ recognition of females’ symptoms. It is important to note, however, that the extent to which this differential presentation and missing diagnoses in females may account for the sex bias in ASD prevalence is not known, and a male bias may very well persist even with increased awareness and adjusted diagnostic criteria.

### Evidence for a female protective effect in ASD

Though ongoing work to better characterize and identify autistic females is required to quantify the true risk-modulatory impact of sex, current data, including general population screens [6, 8–10] and high-risk sibling studies [7, 55, 56], continue to show male-biased prevalence. Furthermore, sex differences in the presentation and experience of ASD symptoms are also consistent with the idea that ASD risk factors have qualitatively, as well as quantitatively, different impact in males and females.

**Fig. 2:**
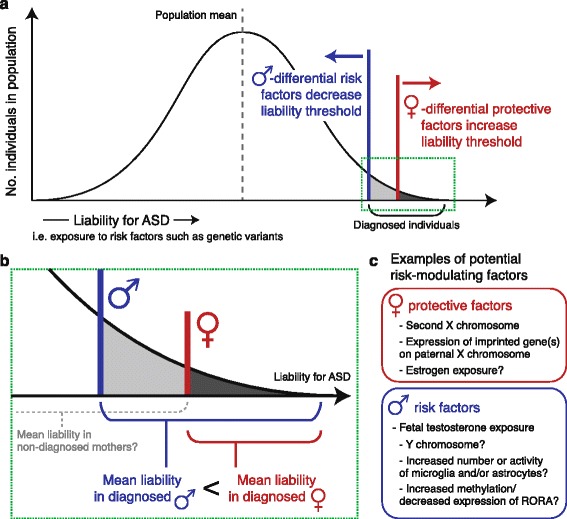
Sex-differential ASD risk can be represented by a multiple-threshold liability/FPE model. **a** Under a multiple threshold liability/female-protective effect (FPE) model, risk or liability for ASD is quantitative and distributed in the population, and males and females have different minimum liability thresholds that are sufficient to lead to an ASD diagnosis. The tail of the distribution filled in *gray* indicates those individuals in the population with diagnosed ASD. **b** A closer look at the region marked with a *green*, *dotted box* in **a**. A key prediction from the FPE model is that among diagnosed individuals, females will have greater ASD liability than males. A secondary prediction is that non-diagnosed females may carry, or be exposed to, relatively high ASD liability but they do not present symptoms that meet criteria for diagnosis; mothers of autistic children may include such females. **c** Examples of sex-differential biological factors proposed to contribute to males’ and females’ shifted liability thresholds and differential risk for ASD. Figure adapted from Werling and Geschwind [46]

One theoretical model for the relationship between sex and ASD risk is derived from a multiple threshold liability model and is commonly referred to in the field as a “female protective effect” (FPE; Fig. 2a) [46, 72]. This model posits that risk for ASD is quantitative, that it follows a distribution in the general population, and that females are protected from the impact of this risk. This female-specific or female-preferential protection leads to a reduced prevalence compared with males. One assumption of this model is of course that, when faced with risk factors, females are protected from becoming autistic. However, as introduced above, it may also be that female-protective mechanisms modulate the effects of risk factors on females’ phenotypes such that they are not diagnosed (i.e., females are protected from *diagnosis* of ASD). Awareness of this possibility is critical and much additional work is required to address it, but for the purposes of exploring current work on the FPE model as it is commonly interpreted, we will accept the assumption that females are protected from ASD itself. Given this, under the FPE model, the threshold of risk burden that females must carry (e.g., deleterious genetic variants) or experience (e.g., environmental exposures), before their neurodevelopment is impacted to the degree that they present with a diagnosable autistic phenotype, is greater than for males.

A key premise of the FPE model is that the factors responsible for the distribution of ASD risk are the same in males and females. It is then hypothesized that female-protective mechanisms act on this common distribution of liability to modulate the impact of risk factors on neurodevelopment and behavior in a sex-differential manner. This is in contrast to a scenario where a subset of risk factors increases ASD risk only in males, which could also lead to sex-differential prevalence. Currently, the full spectrum of risk factors for autism is not understood, but it is well accepted that genetic variation plays a significant role [13–15, 20, 21, 73–78].

With regard to this genetic component of ASD risk, we would expect males and females to be equally likely to carry risk-contributing genetic variants in the same set of genes or at the same loci. In recent years, risk gene discovery work using whole exome sequencing of autism families has dramatically increased the number of genes that can be significantly associated with ASD risk. Apart from ASD-associated monogenic syndromes caused by X chromosome mutations, such as fragile X syndrome, which affects mostly males, and Rett syndrome, which is lethal to males and therefore affects mostly females, these ASD risk loci are predominantly autosomal [15]. Furthermore, analysis of disruptive variants in the 65 genes currently associated with ASD risk demonstrates that these variants are randomly distributed across male and female probands, a pattern that does not support the existence of sex-specific genetic risk factors and is consistent with the premise of a common underlying distribution of genetic risk for ASD in males and females [15].

An important point to make regarding ASD-associated genes and risk loci is that, although the relative risk of disruptive variants in these genes and loci is high, most are not likely to be fully penetrant. This is most clearly seen in studies characterizing the phenotypes of patients and family members who carry copy number variations (CNVs) associated with ASD risk such as 22q11.2 or 16p11.2 deletions. Carriers of the 16p11.2 deletion sometimes do, and sometimes do not, meet diagnostic criteria for ASD, but as a group, carriers show decreased IQ as compared with the general population and with non-carrier family members [79–81], demonstrating that these variants are associated with alterations in neural function in carriers. This and other evidence suggests that genetic background is critical for determining the impact of risk factors, with the additive effects of common variants likely playing a sizeable role in ASD risk [73]. Therefore, it is likely that the same disruptive genetic variant will have different effects on individuals with different genetic backgrounds, pushing individuals over the phenotypic threshold to diagnosable ASD in some cases but not in others [82].

Analogous to the distribution of genetic risk across families and individuals, the behaviors and cognitive patterns that define ASD have also been shown to follow a quantitative distribution in general population samples from the USA [83, 84] and the UK [85, 86]. Idiosyncrasies in social communication and repetitive behaviors or restricted interests are not limited to individuals with ASD, and sub-diagnostic presentation of ASD-like traits, particularly in non-diagnosed family members of ASD cases, are common; this is sometimes referred to as the broader autism phenotype (BAP) [87]. Characterization of quantitative traits associated with ASD has shown that unaffected females in the general population score lower on measures of ASD traits [12, 85, 88] (i.e., more social, more communicative, and less likely to show restricted interests). This basic sex difference, whether driven by innate neurobiology, socialization, or both, may mean that strong risk variants are less likely to push females’ phenotypes into the diagnosable range. However as stated above, in patient cohorts as they are currently ascertained, known ASD-associated genetic variants are randomly distributed between males and females with ASD [15]. Again, this suggests that there is a common set of key genes and loci that modulate ASD risk in both sexes, but that females, on average across the population, are buffered from their effects.

The FPE model also makes several key predictions about the properties and effects of ASD risk factors, each of which can be tested to support or refute the existence of female-differential protection. First and foremost, the FPE model predicts that among diagnosed individuals, females carry or experience greater risk than diagnosed males, on average (Fig. 2b). Given that ASD is highly heritable [48, 55, 89–92], if autistic females carry greater inherited genetic risk than autistic males, then one would expect to observe higher recurrence rates for ASD among the family members of autistic females than males. This pattern of proband sex-mediated recurrence is called the Carter Effect [93], and it has been remarkably difficult to demonstrate in ASD.

Specifically, multiple prospective high-risk sibling studies have failed to find a significant effect of proband sex on ASD recurrence rates in later-born siblings [7, 55]. Though these study designs include families who are likely to be loaded for genetic risk, a relatively small number of families were identified that had both female probands and subsequent affected children, suggesting that these studies may be underpowered to observe the Carter Effect. Interestingly, larger studies on the scale of national registries in Denmark [92] and Sweden [91], which utilized records from over 1.5 million and 2 million children, respectively, also failed to find significantly increased recurrence risk in families with diagnosed females. Both studies tested all combinations of older and younger sibling sex for differences in recurrence rates between these pairings. The study of Swedish children found higher relative recurrence risk in younger siblings of diagnosed females, though this effect did not reach statistical significance. Given the observation of wide confidence intervals around recurrence risk estimates in each group of sex-stratified sibling pairs, the authors of the Danish study cautioned that even their large, non-ascertained cohort included a fairly small number of diagnosed girls and therefore may also be poorly powered to detect significant differences between these groups [92].

In contrast to these reports on high risk and population samples, a study of two population twin cohorts in which ASD traits were measured on a quantitative scale showed significantly higher autistic trait scores in the co-twins of affected females than in the co-twins of affected males [12]. “Affected” here was defined by a score on a quantitative autistic trait measure above the 90th sex-specific percentile, as opposed to a standard diagnosis. This sex-specific quantitative approach for identifying probands is particularly useful, as it has the potential to reduce ascertainment biases against affected females that may result from male-focused diagnostic criteria and screening instruments. Additionally, a study of exclusively multiplex families from the Autism Genetic Resource Exchange (AGRE) cohort reported significantly greater recurrence rates in the later-born children of “female-containing” families (at least one female proband) [94]. In these families from AGRE, recurrence rates were highest for younger male siblings of female probands, suggesting that not only do autistic females carry more penetrant heritable risk for ASD, but that males may be more vulnerable to the inherited risk background that these females share with their siblings.

At present, these disparate results have not been fully reconciled. One reasonable possibility is that differences in genetic architecture between the cohorts used in the studies are responsible for the different patterns observed. Specifically, the Carter Effect is dependent on a penetrant contribution from inherited genetic risk variants that are shared between siblings. Individuals from multiplex families are more likely to carry these inherited risk variants than are simplex, or single incidence families, who show enrichment for risk variants observed only in the child that are not inherited from either parent (*de novo* variation) [14]. Estimates from a volunteer registry and from high-risk sibling studies suggest that only as many as 10–27% of families with an autistic child are multiplex [7, 48, 55, 56]. Therefore, population cohorts and study samples that are not filtered by family type are likely testing children from largely simplex families with primarily *de novo* genetic risk. Since these children are less likely to share these penetrant genetic risk variants with their siblings, this reduces the power to observe significant increases in recurrence in diagnosed females’ siblings.

Bias against the identification of female cases by male-tailored diagnostic instruments may also contribute to this loss of power. If diagnosed females represent a subset of all affected females, and if the key genetic risk variants carried by diagnosed females are more frequently *de novo* than inherited (as compared with diagnosed males), this would reduce the observed recurrence in siblings of female probands. It may be that affected females who currently escape diagnosis are more likely to carry inherited, or common, genetic risk profiles, and identifying these females may improve power to observe significantly higher recurrence in siblings of female versus male probands. However, despite these potential caveats regarding statistical power, the fact that studies of millions of children from national registries do not observe a Carter Effect remains a conundrum.

As discussed above, observing a Carter Effect in a sex-biased condition requires inference about the underlying genetic risk. Today, we can observe most genetic variants directly, including single nucleotide variants (SNVs) and large CNVs or structural variants. In the genetic risk space, then, we can directly test the same prediction of the FPE model that is associated with the Carter Effect: do autistic females carry greater risk than autistic males?

In fact, risk gene discovery studies of *de novo* variants in simplex families do find direct evidence of greater risk burden in diagnosed females at the genetic level. Early work on CNVs in families from the SSC observed a trend toward higher frequency of *de novo* CNVs in autistic females compared with males [13, 14]. These same studies both also found that females’ *de novo* CNVs impact a significantly greater number of genes than those in affected males. A recent analysis of CNVs in the combined SSC and Autism Sequencing Consortium samples was sufficiently powered to observe a statistically significant increase in both the number of genes hit by CNVs and in the frequency of *de novo* CNVs in females [15].

Early exome sequencing studies on the same cohort reported an analogous trend, with a higher rate of *de novo* SNVs overall [16], or exclusively for nonsense [17] or gene disrupting SNVs [18], in female cases. This sex difference, however, did not reach statistical significance in any case. In contrast, a subsequent study of *de novo* indels in SSC cases did observe a significantly increased rate of *de novo* frameshift indels in females [19]. Later analysis of whole exome sequencing in a larger, combined sample of 16 constituent ASD sample sets was able to find a significantly increased rate of *de novo* loss-of-function SNVs in genes associated with ASD risk in females [20]. A simultaneous publication on whole-exome sequencing of the complete SSC further reported that the set of genes disrupted by *de novo* SNVs in females overlaps significantly with the genes disrupted in affected males with low, but not high, IQ [21], demonstrating the high impact of the risk variants that female cases tend to carry.

A potential concern with these findings of greater genetic risk in diagnosed females is the impact of IQ on females’ ascertainment in study cohorts. As discussed, females with cognitive impairment may be more likely to be diagnosed with ASD than those with normal-to-high IQ. Irrespective of sex, ASD cases with low IQ are more likely to carry an identifiable genetic risk variant (e.g., *de novo* loss-of-function SNV, dnLoF, or *de novo* CNV, dnCNV) than cases with higher IQ [15, 21]. Therefore, it may be that the enrichment of disruptive genetic variation seen in female cases is actually a byproduct of the average lower IQ in ascertained female cases as compared with male cases. Work characterizing the phenotypic traits of probands in the SSC has demonstrated that the female probands in this cohort have a lower IQ than the male probands, and that the smaller proportion of females with high IQ (IQ ≥ 80) drives this difference [65]. However, this difference in the distribution of IQ in female probands does not appear to fully account for the observed sex difference in *de novo* risk variant rate. In the analysis of dnCNVs and dnLoF in the combined SSC and ASC cohorts, the presence of a dnLoF or dnCNV was associated with an 8-point decrease in nonverbal IQ (NVIQ) in males and an 18-point decrease in females as compared with sex-matched probands lacking dnLoF or dnCNV [15]. Similarly, both female and male probands with NVIQ below the cohort median of 89 had a significantly higher rate of dnLoF or dnCNVs than sex-matched probands with NVIQ above the median score (females, 2.2-fold enrichment; males 1.6-fold enrichment). Splitting NVIQ into smaller bins (≤50, 51–70, 71–90, 91–110, 111–130, >130) showed that, although the differences did not reach statistical significance, in every NVIQ bin, a greater percentage of female than male probands had a dnLoF or dnCNV. These patterns suggest that the association between female sex and a higher rate of *de novo* risk variants cannot be fully explained by the greater frequency of ascertained females with low IQ in these samples. Taken together, the results of these genetic analyses are in keeping with the prediction from the FPE model that females with autism are subject to greater risk loads than autistic males.

A second prediction of the FPE model mirrors the first: if affected females have greater ASD liability than males, then unaffected females just below the diagnostic threshold are likely to have greater ASD liability than unaffected males as well (Fig. 2b). Put another way, if females are protected from ASD risk, then there are likely to be females in the population who are subject to bona fide ASD risk factors, but who do not present with a diagnosable autistic phenotype. Since the genetic variant space is still the best defined and testable source of ASD risk, screening the general population for ASD risk variants would be one way to identify these high-risk, protected females. However, due to reduced fecundity in ASD [95], interpretable risk variants for ASD are rare, making the required sample size for adequate power in this study design prohibitively high. As sequencing costs drop and commercial and medical genetic testing become more commonplace, such a study may become feasible.

Analogous to the studies of infant siblings of autistic probands, another option is to look at samples of females at high risk for carrying ASD-associated genetic variants. The mothers of autistic cases may represent one such group. Though studies of de novo variants have had great success for identifying ASD risk genes and loci, ASD’s high heritability demonstrates that inherited variation also plays a role in ASD risk. Since mothers of autistic cases are female and therefore experience putative female protective mechanisms that allow them to withstand the impact of ASD risk variants, it has been hypothesized that inherited risk variants in probands are more likely to have come from the mother than the father [72, 96]. A study of transmitted autosomal CNVs in a cohort of individuals with neurodevelopmental disorders and an independent sample of 762 ASD families from the SSC observed exactly this pattern: large, disruptive CNVs over 400 kb in size were more frequently maternally than paternally inherited in both cohorts [11]. More recent analysis of over 2000 families from the SSC corroborates this finding, reporting a significant enrichment in probands compared with siblings for maternally transmitted nonsense SNVs and small CNVs under 100 kb in size [97]. Additionally, a study of copy number genotypes in ASD cases and family members from several sample collections found that mothers in the AGRE cohort carried CNVs that impacted a greater number of genes than fathers’ CNVs, and mothers in the SSC carried a significantly greater number of CNVs than fathers [98].

Given the above support for the FPE model from observed patterns of genetic variation in ASD patients and families, the next aim in the field should be to determine the root cause and mechanism(s) responsible for such a female protective effect. To accomplish this will require both targeted approaches, like the Autism Sisters Project launched by the Autism Science Foundation which will collect data from unaffected sisters of autistic siblings [99], as well as general investigation of the differences between typical male and female neurodevelopment and neural functions.

### Proposed biological risk and protective factors for ASD

Over the years, several preliminary theories for ASD’s sex-differential risk have been proposed and explored. The first, most straightforward hypothesis was that, like many conditions that only affect males, ASD is X-linked [100]. In this scenario, ASD is caused by a mutation in a gene(s) on the X chromosome, and females are protected by their second X chromosome, which carries redundant and likely functional copies of the mutated risk gene(s). In the early days of genetic research in ASD when it was known that ASD showed high heritability but the contributing genetic loci were largely unknown, X-linkage seemed a likely hypothesis. However, due in large part to the rapid and dramatic success of *de novo* variant detection and association approaches, we now know that while there are several ASD-associated genes on the X chromosome, the majority of risk genes are autosomal [15]. Loss-of-function variants in the fraction of risk genes on the X chromosome are not sufficiently common to account for the magnitude of the sex bias in ASD prevalence.

A second hypothesis also invokes the X chromosome, proposing that a single gene on the X that is imprinted and paternally expressed is responsible for females’ protection. Since only females have a paternal X chromosome, males do not express this gene and therefore do not experience its protective properties [40]. This hypothesis is based on observations from a study of individuals with monosomic Turner’s syndrome, which reported that females whose single X chromosome was inherited from their father (45, X^p^) had greater social and executive functioning skills than females with only a maternal X (45, X^m^) [41]. Skuse and colleagues propose that this difference indicates the existence of a locus on the X chromosome that promotes social cognition, and that this locus is only, or largely, expressed from the paternal X. By mapping paternal X chromosome partial deletions in 8 patients with partial monosomy, the researchers concluded that this imprinted, pro-social genetic locus must originate from the q arm of the X, or near the centromere on the p arm. Though intriguing, this locus has not been further resolved, and no single, imprinted, protective gene has been identified to date for ASD.

A third, genetics-based hypothesis proposes that protection in females is conferred by variation at a single genetic locus, whether on the X chromosome or an autosome. This hypothesis was suggested in response to work characterizing quantitative ASD traits in multiplex families; these studies found that scores from the SRS followed a nearly normal distribution across affected and unaffected males, but that females’ scores showed a bimodal distribution split between diagnosed and non-diagnosed individuals [48, 101]. Since in this case, diagnosed and non-diagnosed children are siblings who are assumed to share 50% of a common genetic background, this female-specific bimodal distribution is consistent with the effects of a single, binary locus that can protect carrier females from ASD. However, a sufficiently powered genome-wide association study of 317,574 independent SNPs in 208 female cases and 151 unrelated female controls from AGRE families failed to identify any SNPs significantly associated with ASD status in females [102]. This suggests that the source of the FPE, if it is rooted in genetics, is likely to be polygenic and possibly driven by multiple rare variants instead of a single common variant.

Beyond genetic variation, other proposed mechanisms feature a role for sex steroid hormones during neurodevelopment. The best known is the extreme male brain (EMB) theory, which was proposed by Simon Baron-Cohen and conceptualizes the cognitive-behavioral phenotype of ASD as an amplification of male-typical interests, skills, and behaviors [42]. The theory suggests that there are two key, orthogonal dimensions of sex-differential ability: empathizing, or a drive to perceive and respond appropriately to the thoughts, intentions, and emotions of others, which is more pronounced in females (on average), and systemizing, or a drive to observe and understand the structure and rules of orderly phenomena (math, physics, maps, calendars, mechanics, etc.), which is more pronounced in males. Individuals with autism, then, are those who are especially high systemizers but especially low empathizers.

Since typically developing males, in general, fall closer to the autistic phenotype on these scales than females, Baron-Cohen proposed that testosterone exposure during fetal development may contribute to ASD risk, particularly for autistic girls [42]. Insofar as natural sex differences in testosterone levels may be responsible for amplifying risk in males and/or dialing down risk in females, this hypothesis is consistent with the FPE model. Common thinking about the FPE is that there exists some female-specific, or at least female-preferential, factor or mechanism that actively buffers neurodevelopment against the impact of risk factors. It is important to remember that, while its shorthand name references protection in females, the FPE model is at its root a multiple threshold liability model, and the patterns and predictions of the FPE model are also entirely compatible with the involvement of male-specific risk factors. Such risk factors may operate in lieu of, or in addition to, female-specific protective factors.

Only once a specific mechanism is implicated will we know which is the driving force behind setting males’ and females’ liability thresholds. Until then, whether one considers male-specific risk or female-specific protective factors to be key depends on the baseline of one’s frame of reference. For example, it is straightforward to say that females tend to require more deleterious genetic variants before they present with ASD, and therefore females are protected against lesser variants. This assumes though that the scale of variants’ deleteriousness is based on their impact in males, since females are framed as coping with variants that would typically (i.e., in males) be penetrant. If the deleteriousness of risk variants is instead normalized to their impact in *females*, then one would reinterpret the sex-differential risk variant distribution to mean that it is *males* who require *less deleterious* genetic variants to present with ASD. In this case, the pertinent question is not what mechanism protects females, but what factor or mechanism sensitizes males to this class of less penetrant (in females) risk variants? Since neither males nor females as a group represent the prototypical human, it is not clear which frame of reference is the “correct” one or the one most likely to facilitate discovery of the mechanisms that mediate sex-differential ASD risk. For now, research in this area should consider the possibilities of both female-specific protective factors and male-specific risk factors when designing and interpreting studies.

In their subsequent work to investigate the biology behind the EMB theory, Baron-Cohen and colleagues have performed several studies of the relationship between fetal testosterone levels and phenotypes later in life. In a sample of 235 majority non-autistic children whose mothers had undergone amniocentesis during gestation, the authors observed a significant positive relationship between fetal testosterone levels and quantitative measures of ASD traits [103]. If we conceptualize autistic traits as a continuum in the population that is set by an underlying distribution of exposure to risk factors, then this pattern suggests that testosterone exposure is associated with a shift in the trait distribution toward a diagnosable phenotype. Another study examined the relationship between gestational testosterone levels and neuroanatomy by looking for brain regions with differential gray matter volume in 28 young boys (age 8–11) with different fetal testosterone levels [104]. Here, they observed that regions with testosterone-associated gray matter volume also showed sex-differential gray matter volume in 217 age-matched children with structural imaging data in the National Institutes of Health Pediatric Magnetic Resonance Imaging Data Repository. Specifically, gray matter volume in the right temporoparietal junction/posterior superior temporal sulcus (RTPJ/pSTS) was greater in males and also positively associated with the tested males’ fetal testosterone levels, while gray matter in the planum temporale/parietal operculum (PT/PO) and the posterior lateral orbitofrontal cortex (plOFC) was greater in females and negatively associated with males’ fetal testosterone. Though not directly tied to ASD traits, these findings demonstrate that the putative effects of testosterone exposure align with typical sex differences and that these effects are evident in neuroanatomy. A similar study in girls, particularly those with high testosterone levels (e.g., congenital adrenal hyperplasia patients), would be informative.

Most recently, by making use of national registry data from Denmark, Baron-Cohen and colleagues were able to test for a relationship between fetal testosterone levels and ASD status in the same individuals [105]. By linking biobanked amniotic fluid samples from the gestation of children in the Danish Historic Birth Cohort to records of their later diagnoses in the Danish Psychiatric Central Register, the authors applied a case-control design to test for differences in gestational levels of several hormones: testosterone, progesterone, 17α-hydroxy-progesterone, androstenedione, and cortisol. Hormone levels were compared between affected and unaffected male children only, as there were insufficient numbers of affected females with banked amniotic samples to perform a comparable analysis in female children. All five hormones, including testosterone, showed mean elevation in affected male children as compared to unaffected males, providing the first significant association between fetal testosterone and ASD. Though the distributions of testosterone and the other tested hormones overlap substantially between cases and controls, it is noteworthy that differences in case and control group means are apparent within a sample of exclusively male children. This suggests that testosterone levels, as opposed to a binary-like relative presence or absence of testosterone, may have a positive relationship with ASD risk. Such a binary model may be an oversimplification, but it is the case that the level of testosterone that a fetus is exposed to during gestation differs substantially by sex, as males’ differentiated testes secrete testosterone to drive further sexual differentiation of the body and brain toward male morphology. Therefore, an alternative hypothesis about the relationship between testosterone and ASD risk is that exposure to testosterone above a certain level (e.g., a level sufficient for morphological masculinization) acts to increase risk, and conversely, exposure below this level has no impact on risk. However, as stated above, the findings from this study suggest that relative levels of testosterone, even within males, are associated with ASD risk. A comparable analysis of females, when available, will be informative for further evaluating these possibilities.

Also, though the testosterone findings from this analysis are compelling, the elevation of related hormones in addition to testosterone suggests that the pathways that translate early hormone exposure to an autistic phenotype are multifactorial. That cortisol was one of the elevated hormones suggests that these ASD risk pathways may involve stress responses. Whether the elevation in cortisol results from a response to stress or is a consequence of increased testosterone or other confounding phenomena is not known, and will require further investigation.

What also remains unclear from each of the above studies of fetal testosterone and later phenotypes is whether fetal testosterone acts to skew risk toward males simply because males are more likely to be exposed to higher levels of testosterone or instead whether fetal testosterone preferentially impacts males because females are protected from its effects via some other process. This question is perhaps best addressed by studying the relationship between testosterone levels and ASD-associated phenotypes specifically in females, to determine how testosterone exposure impacts this presumably protected group. Individuals with congenital adrenal hyperplasia (CAH) have a deficiency in the enzyme 21-hydroxylase which causes their adrenal glands to produce abnormally high levels of testosterone, providing a unique population in which to examine the effects of testosterone in females. One study of 34 women with CAH and 24 of their unaffected relatives found that females with CAH scored significantly higher on the Autism Spectrum Quotient (AQ), a self-report questionnaire that measures individuals’ autistic traits [106]. CAH females’ scores were comparable to unaffected males, while males with (*N* = 26) and without (*N* = 25) CAH showed no difference in AQ scores. Despite the small sample size, this finding suggests that, insofar as prenatal exposure to testosterone above a certain threshold shifts individuals’ autistic trait measures toward the diagnosable end of the distribution, females are similarly impacted by testosterone exposure as males.

That testosterone may also affect females’ ASD risk is further supported by reports linking ASD and polycystic ovary syndrome (PCOS), another condition associated with increased testosterone production. In one study, a higher than expected proportion of females with ASD (*N* = 415) reported symptoms of steroid hormone irregularities such as irregular menstrual cycles or precocious puberty, that are frequently associated with PCOS [107]. A more recent analysis of national registry data from Sweden found that children of women with PCOS are at higher risk for ASD (odds ratio (OR) 1.59, 95% confidence interval (CI) 1.34–1.88), and that this risk is further exacerbated by comorbid obesity in mothers, a condition known to be both a contributor and consequence of high androgen levels in PCOS patients (OR 2.13, CI 1.46–3.10) [108]. This study also showed that the magnitude of this increase in risk to offspring of mothers with PCOS was equivalent for male and female children, as compared to children of the same sex whose mothers did not have PCOS (males OR 1.60, CI 1.31–1.94; females OR 1.58, CI 1.14–2.20). Together, these findings are consistent with the hypothesis that elevated exposure to testosterone and/or irregular steroidogenic activity is associated with elevated ASD risk. They also suggest that the source of this exposure may vary, including exposure to the mother’s hormonal state during gestation, or as a result of a comorbid steroidogenic abnormality in the female ASD patient herself.

Under the EMB/fetal testosterone model, the patterns observed in these studies are consistent with the idea that females’ protection and males’ risk is rooted in population-level differences between females’ and males’ exposures to testosterone. Additional research may uncover important nuances in the pathways linking testosterone to ASD neurodevelopment, possibly including androgen receptor regulation in specific cell types or brain regions, or the local generation of androgens from other steroid molecules by populations of brain cells. However, it remains possible that exposure to testosterone, whether systemic or locally generated in the brain, contributes strongly to ASD risk. For example, testosterone may very well initiate or maintain neurodevelopmental processes that steer the brain toward more autistic-like circuitry and function. Therefore, several of the next questions for the field to address involve the specifics of neurodevelopment downstream from testosterone exposure. This, of course, becomes the general study of sexual differentiation and dimorphism of the brain, which is not by any means uniquely relevant to ASD. To better understand sex-biased ASD risk, though, the key will be to determine where, when, and how sex differences in the brain intersect with ASD’s etiological pathways. This endeavor would benefit greatly from the applied expertise of sex differences researchers.

### Investigation of molecular and cellular mechanisms linking sex-differential biology and ASD risk

Thus far, the question of how sex-differential neurobiology interacts with the etiological pathways in ASD at a mechanistic level has only been preliminarily addressed. One group of investigators set out to implicate specific molecular pathways in ASD pathophysiology by looking for gene expression differences in lymphoblastoid cell lines (LCLs) from males with ASD and their unaffected brothers. Genes differentially expressed between siblings showed significant enrichment for genes involved in cholesterol metabolism and androgen biosynthesis pathways [109], and a subset of the affected cases also showed higher testosterone levels than their brothers, together supporting patterns of elevated steroidogenic activity in ASD.

Complementary analyses of differential methylation in LCLs and gene expression in the ASD brain identified two candidate genes with elevated methylation in ASD and decreased expression in the ASD frontal cortex and cerebellum [110]. One of these genes, retinoic acid-related orphan receptor alpha (*RORA*), is a transcription factor that is involved in a sex steroid hormone expression regulatory pathway. Specifically, estrogen and androgen receptor binding sites have been found upstream from *RORA*’s transcription start site and its expression in SH-SY5Y cell culture increases in response to treatment with 17-β estradiol (E2) but decreases in response to dihydrotestosterone (DHT) [111]. *RORA* binding sites have been identified upstream from *CYP19A1* (aromatase), an enzyme that converts testosterone to estradiol, and there is a positive relationship between *RORA* and *CYP19A1* expression, including coordinate reduction of both RORA and CYP19A1 protein in *post mortem* ASD frontal cortex [111]. Several other regulatory targets of RORA show sex-differential expression levels, and the correlation between *RORA* and target expression is stronger in males [112], further linking *RORA* to sex-differential biology. It remains unclear as to whether *RORA* acts as a primary risk factor for ASD, or whether its dysregulation in ASD is a downstream consequence of altered androgen or estrogen expression. Regardless, this deep characterization approach for investigating ASD-associated molecular pathways will be important for understanding the mechanistic links between ASD and sex-differential biology.

Genome-wide screening approaches will also be important for identifying additional molecular and cellular pathways involved in ASD and sex-differential biology. Unlike candidate gene approaches, genome-wide methods such as exome, genome, or even RNA sequencing have the potential to provide an unbiased survey of the key biological processes involved in ASD. A recent study applied such a tactic, using a genome-wide survey of sex-differentially expressed (sex-DE) genes in the adult and fetal human *post mortem* cortex, and results from exome sequencing and coexpression network analyses in ASD, to determine how risk genes or related pathways overlap with sex differences in the human brain [113]. If ASD-associated risk genes are sex-differentially expressed in the typically developing human brain, then variants in these genes might lead to different outcomes in males and females such that a greater proportion of variant-carrying males meet diagnostic criteria for ASD. Alternatively, if ASD-associated risk genes are not sex-differentially expressed, then the risk-modulatory effect of sex must operate on other, interacting molecular pathways. Combining results from genome-wide gene expression studies in ASD and control samples may facilitate the identification of these key molecular points of intersection.

**Fig. 3:**
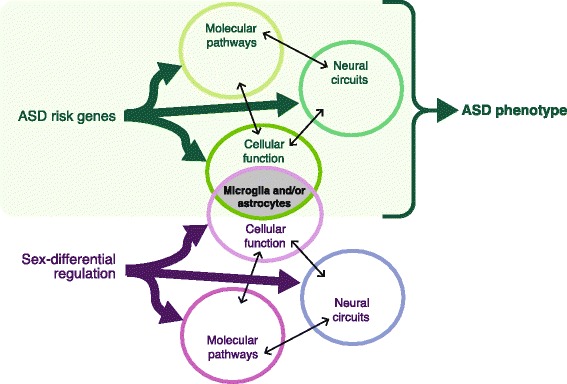
Microglia and/or astrocytes may have a role in ASD pathophysiology and sex-differential biology. ASD risk genes, when disrupted, affect processes in the developing brain such as molecular pathways, cellular functions, and neural circuits (*thick green arrows*), which subsequently lead to an ASD phenotype. Sex-differential regulatory mechanisms also influence different, and possibly overlapping, processes (*thick purple arrows*). A gene recent gene expression analysis by Werling and colleagues (2016) demonstrated that genes associated with the functions of microglia and/or astrocytes show higher expression in males (versus females) as well as higher expression in the ASD brain (versus controls), suggesting that these cell types may contribute to both typical sex differences in the brain and ASD pathophysiology. This is one potential pathway that may contribute to ASD’s male-biased prevalence

In fact, this analysis did not find any evidence for sex-differential expression of ASD-associated risk genes as a class. Instead, the results showed significant overlap between genes with higher expression in males [113] and genes up-regulated in *post mortem* autistic brain relative to similarly male-skewed control groups [114, 115]. These ASD-up-regulated genes that show enrichment for higher expression in males are coexpression modules associated with functions related to the immune system in the brain, microglia, and astrocytes. Genes with higher expression in males also showed significant enrichment for sets of astrocyte and microglial marker genes derived from independent experiments [116, 117]. Together, these patterns suggest that some aspects of ASD biology, particularly functioning of the immune system and/or glial cells, parallels the differences between typical male and female neurobiology (Fig. 3). Currently, evidence exists in the literature for sex differences in rodents in astrocyte morphology in the hypothalamus [118–120], hypothalamic astrocyte responses to estradiol [121, 122], and in microglial colonization of the brain [123, 124], and these new gene expression data from humans indicate that a similar sex difference may exist in the human cortex as well.

Looking forward, given the small number of independent samples in each tested data set, it is imperative that this observation of male-skewed expression of astrocyte and microglial genes be replicated in independent, well-powered data sets. If this pattern does reflect biological truth, it opens up the next round of questions regarding the cause of this sex-differential gene expression and its mechanistic relationship with ASD etiological pathways. For example, is the higher expression of genes associated with astrocyte and microglial functions in males the result of sex differences in cortical cell type composition, i.e., do males have a greater number of microglia than females? Do males’ astrocytes and microglia express marker genes at higher levels? If so, what sex-differential regulatory mechanisms are responsible for directing these differences in expression levels? Or, are other cell types in males more likely to show ectopic expression of astrocyte and microglial marker genes?

Given the overlap at this particular functional and cellular junction with gene expression patterns observed in ASD brain, the answers to these questions are likely to be pertinent for understanding ASD risk and for identifying potential therapeutic targets. Rigorous experimental work to pursue this line of questioning or others that may emerge from high-throughput, bioinformatics approaches would certainly not only be relevant to ASD but would also advance our knowledge of the sex-differential development, structure, and function of the human brain more generally. Concerted and collaborative efforts between ASD clinicians, epidemiologists, geneticists, and neuroscientists with expertise in sex differences will be required to facilitate progress toward better understanding of the processes governing sex-differential neurobiology, ASD biology, and the ways in which they intersect with each other to increase risk for, or protect against, ASD.

## Conclusions

ASD is a condition with a striking male bias in prevalence that remains largely unaccounted for. Though the field is beginning to appreciate that a diagnostic bias against the identification of affected females may contribute, careful prevalence screens that do a better job of identifying affected females so far indicate that ASD may remain approximately twice as prevalent in males. Further investigation is imperative to determine if there is a population of affected, currently undiagnosed females who would benefit from diagnosis and treatment. Additionally, studies of phenotypic differences in autistic males and females show that females present with fewer restricted interests and repetitive behavior symptoms and greater social motivation. It remains to be determined whether these differences result from sex-differential socialization or expectations or whether they are rooted in sex-differential neural circuitry or activity. Studies of genetic risk variants in ASD patients are increasingly consistent with the actions of female protective and/or male risk factors, and investigation of putative molecular and cellular factors suggests that fetal testosterone and/or other steroid hormones, a regulatory pathway involving the gene *RORA*, and sex-differential functioning of astrocytes and microglia may participate in sex-differential risk mechanisms.

Though each of these observations serves to chip away at the gaps in our understanding of the interactions between sex-differential biology and ASD risk pathways, each implicated sex-differential factor has been associated with ASD in relative isolation. Much work remains to flesh out the mechanistic pathways that connect sex-differential biological factors to differences in ASD risk and prevalence. Work that taps into the expertise of the sex differences research community will be critical to accomplish this goal.

In particular, as our list of ASD genes grows, it will be useful to generate and curate similarly robust knowledge of the genes that are involved in sexually dimorphic biology, including genes with sex-differential expression, genes that are regulatory targets of estrogen, androgen, and other sex steroid hormone receptors, and genes that are regulated by transcription factors translated from sex-linked genes. Spatiotemporal information about sex-differential expression and regulation across developmental time, brain regions, and cell types would also increase the utility of such resources by allowing researchers to pinpoint critical points of intersection between sex-differential regulatory pathways and the etiological mechanisms of ASD or other conditions.

It will also be useful to work toward advancing our understanding of gross and fine-scale neurobiological sex differences in brain regions outside of the hypothalamus, a region that has received much experimental attention due to its role in reproductive behavior and robust morphological sex differences. However, ASD and other neuropsychiatric disorders with sex-differential prevalence or presentation largely involve other regions and circuits, and an awareness of where and to what degree sex differences exist throughout the brain will facilitate our understanding of the root causes of these differences. In addition to focused study by sex differences researchers, the newly implemented mandate from the US National Institutes of Health that requires scientists across diverse fields to include sex as a variable in preclinical research may further build on our knowledge of sex effects in a wider array of biological systems. This increased attention to, and awareness of, sex in biological research at all stages will also increase the value of generating a foundational understanding of sex differences in human brain development and function.

Ultimately, the aim of research in this area is to develop a comprehensive understanding of sex in the brain, in development, and in risk mechanisms for ASD. This understanding will be critical, as sex is a potent modulator of ASD risk, and therefore knowledge of the pathways that link sex differential biology to the ASD phenotype may offer key targets for effective, well-tolerated therapeutics. In general, a mutual awareness of the approaches used and findings from sex differences research and of work on ASD genetics and neurobiology will facilitate this intended progress toward making a tangible positive impact on the lives of individuals with autism and their families.

## Abbreviations

ABIDE: Autism Brain Imaging Data Exchange
ADDM: Autism and Developmental Disabilities Monitoring Network
ADI-R: Autism Diagnostic Interview-Revised
ADOS: Autism Diagnostic Observation Schedule
AGP: Autism Genome Project
AGRE: Autism Genetic Resource Exchange
AQ: Autism Spectrum Quotient
ASD: Autism spectrum disorder
ASSQ-REV: Autism Spectrum Screening Questionnaire-Revised
BAP: Broader autism phenotype
CAH: Congenital adrenal hyperplasia
CDC: Centers for Disease Control
CI: Confidence interval
CNV: Copy number variant
DD: Developmental delay
DHT: Dihydrotestosterone
dnCNV: *De novo* copy number variant
dnLoF: *De novo* loss-of-function variant
E2: 17-β estradiol
EMB: Extreme male brain
FPE: Female protective effect
IQ: Intelligence Quotient
LCL: Lymphoblastoid cell line
NDAR: National Database for Autism Research
NHIS: National Health Interview Study
NVIQ: Nonverbal intelligence quotient
OR: Odds ratio
PCOS: Polycystic ovary syndrome
PDD-NOS: Pervasive developmental disorder-not otherwise specified
plOFC: Posterior lateral orbitofrontal cortex
PT/PO: Planum temporale/parietal operculum
RTPJ/pSTS: Right temporoparietal junction/posterior superior temporal sulcus
sex-DE: Sex-differentially expressed
SNV: Single nucleotide variant
SRS: Social Responsiveness Scale
SSC: Simons Simplex Collection
VABS: Vineland Adaptive Behavior Scale
